# The Association between Physical and Psychological Domestic Violence Experienced during the COVID-19 Pandemic and Mental Health Symptoms

**DOI:** 10.3390/ijerph20043312

**Published:** 2023-02-14

**Authors:** Emily M. Lund, Katie B. Thomas

**Affiliations:** 1 Department of Educational Studies in Psychology, Research Methodology and Counseling, University of Alabama, P.O. Box 870231, Tuscaloosa, AL 35487, USA; 2 Clement J. Zablocki VA Medical Center, 10 Tri-Park Way, Appleton, WI 54914-1658, USA

**Keywords:** domestic violence, COVID-19, mental health, post-traumatic stress, depression

## Abstract

Research has shown that rates of domestic violence generally increased during the coronavirus 2019 (COVID-19) pandemic, likely related to mitigation efforts that promoted staying at home and lockdown protocols. However, the link between pandemic-related domestic violence victimization and mental health outcomes has been less explored. The present study examined the possible association between exposure to domestic physical and psychological violence during the COVID-19 pandemic and depressive and post-traumatic stress symptoms (PTSS) in an online sample of American adults recruited in December 2021. Data from 604 participants were analyzed. Forty-four percent of participants (*n* = 266) reported experiencing physical domestic violence, psychological domestic violence, or both during the pandemic, with psychological violence more commonly reported than physical violence. Exposure to both forms of violence was associated with higher rates of depressive and post-traumatic stress symptoms. Given the high rates and negative associations between psychological domestic violence and mental health symptoms in this sample, healthcare providers should assess for domestic violence exposure even if no indications of physical abuse are present or if there were not concerns about domestic violence exposure prior to the pandemic. Potential psychological sequalae should also be assessed if a patient has a positive history of domestic violence victimization.

## 1. Introduction

Domestic violence is a long-standing public health issue [1] Although domestic violence is often considered to be synonymous with “intimate partner violence”, researchers recognize that violence can be perpetrated by other individuals in the home who are not intimate partners (e.g., adult children residing at home, parents, other family members, housemates) [2,3]. Although generally more common in women, it occurs across genders and can occur at any age [2,4]. It can be physical, emotional, sexual, verbal, or financial, and victims often experience more than one type of abuse [4].

Concerns about potential secondary effects around domestic violence began early in the novel coronavirus 2019 (COVID-19) pandemic [3,5,6,7]. Pandemic mitigation efforts strongly encouraged individuals to stay at home whenever possible to reduce the potential spread of COVID-19; this was both a necessary public health action and something that had the potential to place those experiencing or at high risk for domestic violence in situations where they were essentially confined with a perpetrator of abuse for long periods of time, with little or no ability to physically leave the situation or obtain outside support [3,5,6]. Researchers have generally found that rates of domestic violence did appear to increase during the pandemic [8,9,10], with even more pronounced increases seen in North America [9]. Risks were generally more elevated immediately following the implementation of lockdown and similar protocols [8,9] although much of this research cannot account for the various rises and falls in social distancing that occurred throughout the various waves of the COVID-19 pandemic.

Domestic violence has been well documented to be correlated with a variety of negative physical and mental health outcomes [11], including depressive symptoms and post-traumatic stress symptoms (PTSS). Likewise, researchers have found associations between experiencing intimate partner violence during COVID-19 and higher likelihood of severe depressive symptoms [12] although much of this research only examined these associations in the context of the early pandemic (e.g., spring and summer 2020) [9,12], which may not account for the enduring effects of domestic violence on mental health over time or the different “waves” of risk that may have occurred during the pandemic. Research explicitly linking domestic violence victimization and PTSS in the specific context of the COVID-19 pandemic has been limited, although the overall experience of the pandemic itself has been linked to elevated rates of depressive symptoms and PTSS [13,14].

### Purpose of the Present Study

The purpose of the present study is to examine the association between the experience of domestic physical and psychological violence during the COVID-19 pandemic and depressive and post-traumatic stress symptoms (PTSS) in a sample of American adults recruited online. Our research questions are as follows:

1. What was the prevalence of physical and verbal domestic violence victimization during the COVID-19 pandemic in a sample of American adults?
2. How are domestic violence victimization during the COVID-19 pandemic, depressive symptoms, and PTSS related?

## 2. Method

### 2.1. Recruitment and Data Cleaning

Participants were recruited as part of a larger study on traumatic and stressful experiences and mental health during the COVID-19 pandemic that contained items and measures related to demographics, traumatic and stressful experiences, and mental health outcomes. All participants in the study who completed the required items for these analyses (i.e., the measures of depressive symptoms and PTSS and the domestic-violence-related items) and passed the screening checks described below were included in the present analyses. The study was advertised as “an anonymous online survey about experiences during the pandemic, mental health, and well-being”. Although “difficult events” were mentioned in the electronic informed consent, trauma or domestic violence were not specifically mentioned. Recruitment took place in December 2021, during the Delta wave and the beginning of the first Omicron wave of the pandemic in the United States; nationally, most heavy lockdown restrictions had been lifted, and vaccines were widely available, but mask mandates were still commonly in place [15]. Recruitment took place on the Prolific participant recruitment platform (https://www.prolific.co/), which allows approved individuals to anonymously participate in targeted online surveys and other studies online and be compensated for their time.

In order to participate, users had to (a) reside in the United States, (b) be age 18 or older, and (c) successfully complete a series of comprehension questions regarding their rights as participants after reading the informed consent but before beginning the survey. In order to ensure a diverse participant sample, recruitment was conducted in a series of four waves based on racial/ethnic identity (White, non-Hispanic or non-White) and disability status (has a disability or chronic health condition or does not) using Prolific’s targeted recruitment options. All participants completed the same questionnaire regardless of recruitment wave, no participant could complete the survey multiple times, and data from all four waves were combined for analytic purposes. Participants were compensated USD 3.50 for their participation, and no identifying information was collected. All procedures were approved by the lead author’s institutional review board (IRB) before data collection began.

Six hundred and sixty-one participants completed the survey. Of these, three participants (0.6%) were removed for failing one or both of the two attention check items embedded in the survey. These items required participants to select a specified response and are often used in online survey research to help reduce bots and random clicking [16], as were the comprehension items that had to be successfully completed in order to be begin the survey. An additional 12 participants (2.4%) were removed for giving different responses to one repeated item, indicating potential poor attention to the items. Additionally, qualitative items in the survey served as an informal check for participant attention and quality [17]; responses to these items tended to be logical and detailed, suggesting high levels of participant attention. Of the 646 remaining participants, 1 (0.2%) was removed for not completing the items related to domestic violence, 19 (2.9%) were removed for missing depressive symptom scores, and 26 (4.0%) were removed for missing post-traumatic stress symptom scores, with 4 missing scores for both depressive and post-traumatic stress symptom measures. In all, a total of 42 participants were removed for missing data. Thus, the final sample for the present analyses was 604.

### 2.2. Participants

Of the 604 included participants, most (65.7%; *n* = 397) identified as female, with 186 (30.8%) identifying as male and 21 (3.5%) identifying outside the gender binary (e.g., agender, non-binary). In terms of sexual orientation, most participants (67.1%; *n* = 405) identified as heterosexual, 129 (21.4%) as bisexual or pansexual, 39 (6.5%) as gay or lesbian, 23 (3.8%) as asexual, and 8 (1.3%) as another sexual orientation. Approximately one-quarter of participants (24.8%; *n* = 150) identified as having a disability, and 35.1% (*n* = 212) identified as having a chronic health condition. These items were not mutually exclusive.

In terms of race and ethnicity, 247 participants (40.9%) identified as White/Caucasian, 100 (16.6%) as Asian/Pacific Islander, 99 (16.4%) as Hispanic/Latino, 92 (15.2%) as African-American/Black, 26 (4.3%) as Native American/Alaskan Native, and 18 (3.0%) as Arab/Middle Eastern. Thirty-six participants (6.0%) selected the “other” option, and 40 (6.6%) indicated that they did not wish to provide their race or ethnicity. Participants could choose multiple options for their race/ethnicity.

In terms of relationship status, two-fifths (*n* = 250 41.4%) of participants were single, 31 (5.1%) were divorced, 5 (0.8%) were separated, and 2 (0.3%) were widowed. Furthermore, 100 participants (16.6%) were dating, 72 (11.9%) were unmarried but living with a partner, and 130 (21.5%) were married. In terms of employment, 244 participants (40.4%) were working full-time, 98 (16.2%) were working part-time, and 89 (14.7%) were full-time students. One hundred and forty-nine (24.7%) were unemployed. In terms of education, 291 (48.2%) had a bachelor’s degree or higher, 64 (10.6%) had an associate’s degree, and 168 (27.8%) had some college.

### 2.3. Measures

All measures were completed via a survey hosted on a secure, university-based Qualtrics server.

#### 2.3.1. Demographics

Participants were asked to answer questions regarding race/ethnicity, disability status and type(s) of disability, sexual and romantic orientation, employment status (including recency of employment), income range, marital status, and other variables.

#### 2.3.2. Physical and Psychological Domestic Violence

The abuse-related items were part of a pandemic-related stress and trauma measure was specifically created for this study. Participants were asked to rate how often during the pandemic they experienced each item on a scale of 1 (“never”) to 5 (“very often”). Two items relating to domestic violence victimization were used in the present analyses. The physical domestic violence item asked participants if and how often they were “hit, kicked, punched, or otherwise physically hurt by a family member, significant other, housemate, or caregiver”. The psychological domestic violence item asked participants if and how often they were “yelled, insulted, or screamed at by a family member, significant other, housemate, or caregiver”. Because of the lack of variance in response distribution for these items (see Results Section), they were largely dichotomized (i.e., experienced abuse or did not experience abuse) for analytic purposes.

#### 2.3.3. Depressive Symptoms

Depressive symptoms were measured using the Center of Epidemiological Studies Depression Scale (CESD) [18]. The CES-D consists of twenty items asking about participants’ experiences of common symptoms of depression over the last seven days; each item is scored on a four-point scale from 0 (one day or less than one day) to 3 (5–7 days). Possible scores range from 0–60, and 16 has been recommended as a cut-off score for determining likely major depressive disorder [18]. The CES-D has been shown to be a valid screening measure for detecting depressive symptoms [19] and has demonstrated acceptable internal consistency for both general (*α* = 0.85) and clinical (*α* = 0.90) samples [18]. The Cronbach’s *α* in the present sample was 0.94. Four hundred and two participants (66.6%) of the present sample met or exceeded the suggested cut-off of 16 for likely major depression, and the mean score for the entire sample was 23.10 (SD = 13.67; range = 0–59).

#### 2.3.4. Post-Traumatic Stress Symptoms

Post-traumatic stress symptoms (PTSS) were measured using the Patient Checklist-5 (PCL-5) [20]. It asks about participants’ experiences of being bothered by 20 different symptoms of post-traumatic stress over the past month. Each item is rated from 0 (“not at all”) to 4 (“extremely”) for a possible range of 0–80. The PCL-5 is well validated and has demonstrated good sensitivity, specificity, and reliability [20]. In order to orient participants’ responses to PTSS related to pandemic-related stressors specifically, participants were asked to think about only symptoms related to the pandemic-related stressors asked about on the pandemic stress and trauma scale. The Cronbach’s *α* in the present sample was 0.96.

A total score cut-off of 31–33 is recommended for determining likely post-traumatic stress disorder [20]; we used a cut-off of 33 to be conservative in our estimates. Two hundred participants (33.1%) met or exceeded the cut-off of 33 for likely post-traumatic stress disorder, and the mean score for the total sample was 24.56 (*SD* = 19.71; range = 0–80).

### 2.4. Data Analysis

For the first research question, simple descriptive statistics were used to report the number and percentage of participants who reported experiencing domestic violence during the pandemic. As noted previously, these items were largely dichotomized (i.e., reported experiencing abuse or did not report experiencing abuse) for statistical analyses, as the strong majority of respondents (76.6–90.1%) reported experiencing each type of abuse only “once” or “a few times” during the pandemic.

For the second research question, independent-sample *t*-tests were used to assess differences in mean reported symptom levels for participants who did or did not report experiencing certain types of domestic violence during the pandemic. Cohen’s *d* effect sizes were also reported for these analyses; standard cut-offs for small (0.2), medium (0.5), and large (0.8) effect sizes were used [21]. For analyses concerning the percentage of participants reporting likely diagnostic levels of symptoms, chi-square analyses were used. Phi effect sizes were also reported, again using the standard cut-off points of 0.1 for small, 0.3 for medium, and 0.5 for large effect sizes [21]. For each outcome variable (i.e., depressive symptoms or PTSS), the following comparisons were conducted: (a) any domestic violence vs. no domestic violence, (b) psychological domestic violence only vs. no domestic violence, (c) physical domestic violence vs. no domestic violence, and (d) both physical and psychological domestic violence vs. only one type of violence. Because only one participant reported experiencing physical domestic violence in the absence of psychological domestic violence, we removed that participant from analyses in order to test if their inclusion or exclusion made a difference in the results. Because the results remained essentially unchanged either way, we elected to retain the participant in the interest of completeness.

## 3. Results

### 3.1. Prevalence of Domestic Violence

Over two-fifths of the participants (44%; *n* = 266) reported experiencing either physical domestic violence or psychological domestic violence or both during the pandemic. Psychological domestic violence was considerably more common and was experienced by (43.9%; *n* = 265) of participants. In contrast, only 74 participants (12.3%) reported experiencing physical domestic violence during the pandemic. Seventy-three participants (12.1%) experienced both physical and psychological violence, one (0.2%) experienced physical violence only, and one hundred and ninety-two (31.8%) experienced psychological domestic violence only. The remaining 338 participants (56%) reported experiencing neither physical nor psychological domestic violence during the pandemic.

The frequency of reported domestic violence victimization was towards the lower end of the scale (i.e., “once” or “a few times”) for both physical and psychological abuse. Of the 74 participants reporting physical domestic violence victimization, 33 (44.6%) reported experiencing it once and 34 (45.9%) a few times. The remaining seven reported experiencing it often (4.1%; *n* = 3) or very often (5.4%; *n* = 4). Of the 265 participants who reported experiencing psychological domestic violence, 41 (15.5%) reported experiencing it once, 162 (61.1%) a few times, 48 (18.1%) often, and 14 (5.3%) very often.

### 3.2. Association between Domestic Violence and Depressive Symptoms

Participants who reported experiencing domestic violence reported significantly higher levels of depressive symptoms (*M* = 28.30, *SD* = 12.94) than did those who did not report experiencing domestic violence (*M* = 19.00, *SD* = 12.83) (*t*(602) = 8.815, *p* < 0.001, *d* = 0.732). This was also true for those who had experienced psychological abuse only compared to those who had not experienced domestic violence (*M* = 27.23; *SD* = 13.20 vs. *M* = 19.00; SD = 12.83) *(t*(528) = 7.379, *p* < 0.001, *d* = 0.667). The same pattern also held when only the experience of physical abuse was considered (*M* = 31.11; *SD* = 11.85 vs. *M* = 19.00; *SD* = 12.83) (*t*(410) = 7.448, *p* < 0.001; *d* = 0.956). Participants who reported experiencing both physical and psychological domestic violence had significantly higher levels of depressive symptoms than those who reported experiencing only one form of abuse (*M* = 31.12; *SD* = 11.93 vs. *M* = 27.24; *SD* = 13.17) (*t*(264) = 2.198, *p* = 0.029, *d* = 0.302).

In terms of the meeting the criteria for likely major depression, 82.0% of those who reported experiencing domestic violence met or exceeded the CES-D cut-off score of 16 compared to 54.4% of those who did not. This difference was significant (χ(1) = 50.636, *p* < 0.001, ϕ = 0.290). The percentage of participants meeting the clinical cut-off for depression was also significantly higher among those who reported experiencing only psychological domestic violence than those who did not report experiencing domestic violence (78.6% vs. 54.4%; χ(1) = 30.855, *p* < 0.001, ϕ = 0.241) and when those reporting physical domestic violence were compared to those not reporting domestic violence (90.5% vs. 54.4%; χ(1) = 33.237, *p* < 0.001, ϕ = 0.284). Finally, participants who reported experiencing both physical and psychological domestic violence during the pandemic were more likely than participants who reported experiencing only one type of domestic violence to report depressive symptoms at or above the cut-off point for likely major depression (90.4% vs. 78.7%; χ(1) = 4.865, *p* = 0.027, ϕ = 0.135).

### 3.3. Association between Domestic Violence and PTSS

Participants who reported experiencing any domestic violence reported significantly higher levels of PTSS than did those who did not report experiencing domestic violence during the pandemic (*M* = 32.62; *SD* = 19.42 vs. *M* = 18.22; *SD* = 17.51) (*t*(539.13) = 9.438, *p* < 0.001, *d* = 0.782). This was also true for participants who experienced only psychological abuse (*M* = 30.252; *SD* = 18.93 vs. *M* = 18.22; *SD* = 17.51) (*t*(528) = 7.379, *p >* 0.001, *d* = 0.667). Likewise, levels of PTSS were higher when only physical abuse was accounted for (*M* = 38.74; *SD* = 19.45 vs. *M* = 18.22; *SD* = 17.51) (*t*(401)) = 8.944, *p* < 0.001; *d* = 1.184). Finally, levels of PTSS were also significantly higher in individuals who reported experiencing both physical and psychological domestic violence during the pandemic versus those who only reported experiencing one type of abuse (*M* = 38.73; *SD* = 19.59 vs. *M* = 30.31; *SD* = 18.90) (*t*(264) = 3.210, *p* < 0.001, *d* = 0.441).

Participants who reported experiencing domestic violence during the pandemic were significantly more likely than those who did not to report PTSS that fell within the range indicating likely post-traumatic stress disorder (47.0% vs. 22.2%; χ(1) = 41.346, *p* < 0.001, ϕ = 0.262). Participants who experienced only psychological domestic violence were also significantly more likely than those who did not experience domestic violence during the pandemic to have PTSS scores that met the cut-off for likely post-traumatic stress disorder (41.7% vs. 22.2%; χ(1) = 22.449, *p* < 0.001, ϕ = 0.206) The association between experiencing domestic violence and having PTSS at or above the cut-off was also true when only physical abuse was taken into account (60.8% vs. 22.2%; χ(1) = 43.867., *p* < 0.001, ϕ = 0.326). Finally, participants who reported experiencing both physical and psychological domestic violence during the pandemic were more likely than participants who reported experiencing only one type of domestic violence to have PTSS at or above the cut-off point for likely post-traumatic stress disorder (60.3% vs. 42.0%; χ(1) = 7.125, *p* = 0.008, ϕ = 0.164).

## 4. Discussion

In the present study, we examined the prevalence of self-reported physical and psychological domestic violence victimization during the first year and half of the COVID-19 pandemic and its association with depressive and post-traumatic stress symptoms in a sample of American adults. Psychological domestic violence victimization was considerably more common than physical domestic violence victimization in our sample, and all but one participant who reported experiencing physical violence also reported experiencing psychological violence. Both physical and psychological violence were associated with higher levels of depressive symptoms and PTSS, and participants who experienced domestic violence during the pandemic were more likely to have a level of psychological symptoms suggestive of a depressive or trauma-related disorder. Effect sizes were generally in the small-to-medium to medium range for symptom scores and in the small to small-to-medium range for the likelihood of meeting clinical cut-offs; this can be expected given the stricter criteria inherent in a clinical cut-off.

Of note, our present study adds to the literature by assessing COVID-19-related domestic violence victimization over a relatively long timespan (approximately 18 months). Much of the existing research on domestic violence during the pandemic is exclusive to the early pandemic or initial lockdowns [8,9]. However, the pandemic has been and continues to be a long-lasting and varying source of both medical and psychosocial stressors that may increase psychosocial vulnerability, particularly among marginalized and already vulnerable groups [22,23]. Thus, some individuals may have experienced domestic violence outside of the first wave of pandemic response and thus may have been accurately captured in our dataset even if they experienced violence outside of the first wave of COVID-19 restrictions. Additionally, the fact that domestic violence was not something that participants reported experiencing “often” during the pandemic but still was associated so strongly with depressive symptoms and PTSS highlights the fact that the psychological impact of domestic violence experienced during the pandemic may reach far beyond the time at which it occurred.

The association of psychological victimization with both physical violence and more severe mental health symptoms is notable and consistent with research from other countries that highlights the high prevalence of psychological domestic violence during the early pandemic [24]. Our findings also highlight the critical importance of asking about psychological violence victimization in clinical settings both when physical victimization is suspected or confirmed and when it is not. Often times, domestic violence may not be inquired about until a major physical injury has occurred [25], meaning that serious psychological violence victimization may be missed in clinical assessment. Psychological violence may both perpetuate and facilitate current or future physical violence [3,26] and cause considerable psychological morbidity alone [27], making proper identification and intervention for it a critical clinical activity.

## 5. Limitations and Directions for Future Research

In interpreting our findings, it is important to note that rates of both domestic violence (44%) and likely major depression (66.9%) and post-traumatic stress disorder (33.1%) were high in the present sample, suggesting that our sample may represent a particularly vulnerable population. This aligns with previous research studies where participants recruited from online sources tend to have higher base rates of clinically notable psychological symptoms than what may be expected in a general population sample [28,29]. Additionally, the rates of both domestic violence and clinically significant psychological symptoms may also be influenced by the purposeful oversampling of individuals with disabilities and chronic health conditions, who are at higher risk for both violence victimization [4] and depression [28]. Thus, these rates may be higher than those found in true random population samples and should not be taken as an overall population prevalence rate.

Additionally, because the strong majority of participants who reported experiencing domestic violence during the pandemic reported only experiencing it “once” or “a few times”, we did not have enough variance in reported domestic violence victimization frequency in the sample to analyze how difference in the frequency of domestic violence victimization during the pandemic relates to depressive symptoms or PTSS. Targeted recruitment specifically of individuals who experienced a high rate of domestic violence during the pandemic may be a helpful way to address this gap in future research.

Another limitation of the present study is that we only asked about physical and psychological domestic violence. Thus, we did not examine the relationships between other forms of domestic violence, such as sexual and financial abuse, and mental health outcomes during the pandemic. Researchers should continue to study domestic violence and its effects in the context of the COVID-19 pandemic and to analyze the varying topographies of abuse in this context. Likewise, future researchers may wish to collect data on the specific perpetrator (e.g., intimate partner, other family member, housemate) in order to better ascertain how different relational dynamics influence the nature and experience of abuse.

Furthermore, we did not ask about pre-pandemic or lifetime domestic violence victimization in the present study, limiting our ability to discuss trajectories of violence before and across the pandemic and the impact of those trajectories on mental health outcomes. Researchers may want to address these questions in future studies. However, we did ask participants to limit their reporting of PTSS to symptoms related to events that occurred during the pandemic. Finally, studying exactly when the domestic violence occurs in the context of the pandemic may be important given previous research findings that indicate that domestic violence risk may be particularly high at the beginning of a lockdown cycle [8,9]. Future research could examine if domestic violence victimization spikes mirrored pandemic spikes, as the latter typically led to increased pressure or necessity to stay at home and thus potentially increased exposure to perpetrators of domestic violence. “Safer at home” orders during pandemics may be somewhat of a double-edged sword for individuals experiencing or at risk for domestic violence; although such actions are helpful in reducing the risks of virus exposure and transmission, especially in vulnerable populations, the isolation may also increase risk of exposure to perpetrators, increase barriers to seeking help, and create potential for psychological vulnerabilities and sequalae [23]. Thus, public health responses should incorporate considerations of domestic violence and strategies for working with those populations for whom home might not be “safe” [5,6].

## 6. Conclusions

This study examines domestic violence victimization and its mental health effects for approximately the first 18 months of the COVID-19 pandemic. We found high rates of psychological domestic violence in our sample, and both physical and psychological violence victimization were associated with higher rates of depressive and post-traumatic stress symptoms. Our findings highlight the importance of assessing for domestic violence exposure, including psychological violence, that may have occurred during the pandemic and as well as identifying its potential psychological sequalae.

## Data Availability

Data are not publicly available due to the sensitive nature of the subject matter and the need to preserve participant anonymity and confidentiality. Sections of the data may be obtained in accordance with IRB approval by contacting the first author in order to verify the results.

## References

[1] Nicolaidis C. (2002). The voices of survivors documentary. J. Gen. Intern. Med..

[2] Lund E.M., Oschwald M., Latorre A., Hughes R.B., Liston B., Shelton R., Flaherty M.C., Porcher E.M., Powers L.E. (2015). Development of the Internet-Based Safer and Stronger Program for Men With Disabilities. Rehabil. Couns. Bull..

[3] Lund E.M. (2020). Interpersonal violence against people with disabilities: Additional concerns and considerations in the COVID-19 pandemic. Rehabil. Psychol..

[4] Hughes R.B., Lund E.M., Gabrielli J., Powers L.E., Curry M.A. (2011). Prevalence of interpersonal violence against community-living adults with disabilities: A literature review. Rehabil. Psychol..

[5] Froimson J.R., Bryan D.S., Bryan A.F., Zakrison T.L. (2020). COVID-19, Home Confinement, and the Fallacy of “Safest at Home”. Am. J. Public Health.

[6] Gabrielli J., Lund E. (2020). Acute-on-chronic stress in the time of COVID-19: Assessment considerations for vulnerable youth populations. Pediatr. Res..

[7] Moreira D.N., da Costa M.P. (2020). The impact of the COVID-19 pandemic in the precipitation of intimate partner violence. Int. J. Law Psychiatry.

[8] Kourti A., Stavridou A., Panagouli E., Psaltopoulou T., Spiliopoulou C., Tsolia M., Sergentanis T.N., Tsitsika A. (2021). Domestic violence during the COVID-19 pandemic: A systematic review. Trauma Violence Abus..

[9] Piquero A.R., Jennings W.G., Jemison E., Kaukinen C., Knaul F.M. (2021). Domestic violence during the COVID-19 pandemic—Evidence from a systematic review and meta-analysis. J. Crim. Justice.

[10] Boserup B., McKenney M., Elkbuli A. (2020). Alarming trends in US domestic violence during the COVID-19 pandemic. Am. J. Emerg. Med..

[11] Nicolaidis C., Curry M., McFarland B., Gerrity M. (2004). Violence, mental health, and physical symptoms in an academic internal medicine practice. J. Gen. Intern. Med..

[12] Iob E., Frank P., Steptoe A., Fancourt D. (2020). Levels of Severity of Depressive Symptoms Among At-Risk Groups in the UK During the COVID-19 Pandemic. JAMA Netw. Open.

[13] Coloma-Carmona A., Carballo J.L. (2021). Predicting PTSS in general population during COVID-19 pandemic: The mediating role of health anxiety. J. Affect. Disord..

[14] Vindegaard N., Benros M.E. (2020). COVID-19 pandemic and mental health consequences: Systematic review of the current evidence. Brain Behav. Immun..

[15] Centers for Disease Control and Prevention (2022). COVID-19 Museum Timeline. https://www.cdc.gov/museum/timeline/covid19.html.

[16] Bogart K.R., Rottenstein A., Lund E.M., Bouchard L. (2017). Who self-identifies as disabled? An examination of impairment and contextual predictors. Rehabil. Psychol..

[17] Thomas K.B., Lund E.M., Bradley A.R. (2015). Composite Trauma and Mental Health Diagnosis as Predictors of Lifetime Nonsuicidal Self-Injury History in an Adult Online Sample. J. Aggress. Maltreat. Trauma.

[18] Radloff L.S. (1977). The CES-D Scale: A self-report depression scale for research in the general population. Appl. Psychol. Meas..

[19] Weissman M., Sholomskas D., Pottenger M., Prusoff B.A., Locke B.Z. (1977). Assessing depressive symptoms in five psychiatric populations: A validation study. Am. J. Epidemiol..

[20] Weathers F.W., Litz B.T., Keane T.M., Palmieri P.A., Marx B.P., Schnurr P.P. (2013). The PTSD Checklist for DSM-5 (PCL-5). http://www.ptsd.va.gov/professional/assessment/adult-sr/ptsd-checklist.

[21] Cohen J. (1992). A power primer. Psychol. Bull..

[22] Lund E.M., Ayers K.B. (2022). Ever-changing but always constant: “Waves” of disability discrimination during the COVID-19 pandemic in the United States. Disabil. Health J..

[23] Lund E.M., Forber-Pratt A.J., Wilson C., Mona L.R. (2020). The COVID-19 pandemic, stress, and trauma in the disability community: A call to action. Rehabil. Psychol..

[24] Jung S., Kneer J., Krüger T.H.C. (2020). Mental Health, Sense of Coherence, and Interpersonal Violence during the COVID-19 Pandemic Lockdown in Germany. J. Clin. Med..

[25] Nicolaidis C., Curry M.A., Ulrich Y., Sharps P., McFarlane J., Campbell D., Gary F., Laughon M.K., Glass N., Campbell J. (2003). Could we have known? A qualitative analysis of data from women who survived an attempted homicide by an intimate partner. J. Gen. Intern. Med..

[26] Lagdon S., Armour C., Stringer M. (2014). Adult experience of mental health outcomes as a result of intimate partner violence victimisation: A systematic review. Eur. J. Psychotraumatol..

[27] Copel L.C. (2006). Partner abuse in physically disabled women: A proposed model for understanding intimate partner violence. Perspect. Psychiatr. Care.

[28] Lund E.M., Nadorff M.R., Galbraith K., Thomas K.B. (2019). Comparing the internal consistency, overall scores, and response patterns on the Suicidal Behavior Questionnaire–Revised in people with and without disabilities. Rehabil. Couns. Bull..

[29] Shapiro D.N., Chandler J., Mueller P.A. (2013). Using Mechanical Turk to Study Clinical Populations. Clin. Psychol. Sci..

